# Microbial Community Analysis of Saliva and Biopsies in Patients With Oral Lichen Planus

**DOI:** 10.3389/fmicb.2020.00629

**Published:** 2020-05-05

**Authors:** Xuewei Wang, Zhibai Zhao, Nan Tang, Yuping Zhao, Juanyong Xu, Liuyang Li, Ling Qian, Junfeng Zhang, Yuan Fan

**Affiliations:** ^1^Jiangsu Key Laboratory of Oral Diseases, Nanjing Medical University, Nanjing, China; ^2^Department of Oral Medicine, Affiliated Hospital of Stomatology, Nanjing Medical University, Nanjing, China; ^3^Medical School, Nanjing University of Chinese Medicine, Nanjing, China

**Keywords:** OLP, 16S rDNA gene, saliva, tissue, microbiome, fluorescence *in situ* hybridization

## Abstract

The specific etiology and pathogenesis of oral lichen planus (OLP) remain elusive, and microbial dysbiosis may play an important role in OLP. We evaluated the saliva and tissue bacterial community of patients with OLP and identified the colonization of bacteria in OLP tissues. The saliva (*n* = 60) and tissue (*n* = 24) samples from OLP patients and the healthy controls were characterized by 16S rDNA gene sequencing and the bacterial signals in OLP tissues were detected by fluorescence *in situ* hybridization (FISH) targeting the bacterial 16S rDNA gene. Results indicate that the OLP tissue microbiome was different from the microbiota of OLP saliva. Compared with the healthy controls, *Capnocytophaga* and *Gemella* were higher in OLP saliva, while *Escherichia*–*Shigella* and *Megasphaera* were higher in OLP tissues, whereas seven taxa, including Carnobacteriaceae, Flavobacteriaceae, and *Megasphaera*, were enriched in both saliva and tissues of OLP patients. Furthermore, FISH found that the average optical density (AOD) of bacteria in the lamina propria of OLP tissues was higher than that of the healthy controls, and the AOD of bacteria in OLP epithelium and lamina propria was positively correlated. These data provide a different perspective for future investigation on the OLP microbiome.

## Introduction

Oral lichen planus (OLP) is a common oral mucosa disease characterized by chronic inflammation, mainly affecting the buccal mucosa, tongue, gingiva, and lower lips (Scully and Carrozzo, 2008). Females between 30 and 60 years are more vulnerable to OLP, which affects 0.5–2% of the general population (Alrashdan et al., 2016; Olson et al., 2016). According to different clinical features, OLP can be classified into two clinical subtypes: reticular OLP and erosive OLP. In histopathology, OLP is mainly characterized by liquefaction degeneration of epithelial basal keratinocytes and band-like layer infiltration of subepithelial lymphocytes (Olson et al., 2016). OLP has a certain malignant potential and its malignant transformation rate is 0.1–2% (Crincoli et al., 2011); the World Health Organization labeled it as a potentially malignant disorder (Tampa et al., 2018).

The etiology and pathogenesis of the OLP are still poorly understood, but they probably involve multiple factors such as infections, autoimmunity, stress, drugs, and so on (Mostafa and Tarakji, 2015). Besides that, oral hygiene, such as plaques and calculus, will also aggravate the occurrence and development of OLP (Crincoli et al., 2011). Oral microbial community plays an increasingly important role in human oral and systemic health (Gao et al., 2018), and microbial dysbiosis may induce or cause chronic inflammation and infection. Studies have shown that OLP may be concerned with viruses, *Candida*, and *Helicobacter pylori* infection (Farhi and Dupin, 2010; Masaki et al., 2011). Recently, a large number of studies have confirmed that the microbial infection is a potential trigger or facilitator of the pathogenesis of human autoimmune disease; the commensal bacteria may participate in local and systemic immune response processes and destroy the normal immune mechanism of the body, such as rheumatoid arthritis, multiple sclerosis, Sjögren’s syndrome, etc. (Alexander et al., 2014). Currently, non-invasive microbial sampling of the OLP, including the collection of saliva and swab samples, is relatively quick and easy to obtain, but it is unclear whether these methods are a valid surrogate for OLP tissue biopsy.

A recent study showed that the overall structure of the salivary microbial community was not significantly affected by the disease status, and the relative abundance of *Porphyromonas* and *Solobacterium* in erosive OLP was significantly higher (Wang et al., 2016). He et al. (2017) reported that the relative abundance of *Fusobacterium*, *Leptotrichia*, and *Lautropia* on the surface of the buccal mucosa of OLP patients was significantly higher, while that of *Streptococcus* of the healthy controls was significantly higher. Besides, Yun et al. (Choi et al., 2016) found that *Streptococcus* decreased in the mucosal surface of OLP patients, pointed out that bacteria could colonize the lamina propria of OLP tissue, and proposed that intracellular bacteria in the tissue may trigger T cell infiltration and provide targeted antigen. However, the above samples typically have lower sensitivity or specificity than tissue samples, and the species of microbiota detected in OLP tissues have not been completely elucidated.

Few investigations to date have attempted to study bacteria in OLP thoroughly. To our knowledge, no more data are available to characterize the local microbiome in OLP tissue samples, which is a critical step to well understand whether and how bacteria play a role in the development of OLP. Most previous studies on OLP microbial community were limited to the collection of saliva and mucosal surface swabs of OLP patients. Due to different colonization conditions of oral mucosal bacteria in different sites, it is often not representative to collect single site samples when OLP lesions occur in multiple sites of the oral mucosa. Therefore, we analyzed the microbial composition and community diversity of tissue samples from OLP patients who underwent pathological biopsy and saliva samples from non-invasive and low-risk methods.

This study aimed to evaluate the microbial communities of different sample types and whether there are differences between them through bacterial 16S rDNA gene amplification sequencing, to analyze the role of the microorganisms with high abundance co-enrichment in the development of OLP of the two sample types, and to locate the bacteria in OLP tissues by fluorescence *in situ* hybridization (FISH). This could provide experimental data for future studies on the potential correlation between saliva and tissue microbiome of OLP patients and healthy people.

## Materials and Methods

### Ethics Statement

This study was approved by the Ethical Committee of the Affiliated Hospital of Stomatology, Nanjing Medical University (permission number PJ2016−034−001) and the Institutional Review Board of Nanjing Medical University (permission number 2014−132). Written informed consent was obtained from each participant and all procedures were conducted following the Declaration of Helsinki.

### Study Subjects

In this study, saliva samples were collected from 40 OLP patients (20 reticular OLP and 20 erosive OLP) and 20 healthy controls. Tissue samples were collected from 24 OLP patients (12 reticular OLP and 12 erosive OLP) and eight healthy controls. All subjects were enrolled from the Department of Oral Medicine of the Affiliated Hospital of Stomatology at Nanjing Medical University from November 2017 to May 2019. Based on the clinical and histopathological diagnostic criteria for OLP proposed by the World Health Organization in 1978 (Kramer et al., 1978) and the criteria set forth by van der Meij and van der Waal (2003), all subjects met the above criteria. The demographic and clinical data of all subjects are shown in Tables 1, 2.

**TABLE 1:** Demographic and clinical parameters of the saliva samples found no statistically significant differences in age (*P* = 0.526 b) and gender (*P* = 0.471 a) of each group.

Characteristics	Healthy controls (*n* = 20)	Reticular OLP patients (*n* = 20)	Erosive OLP patients (*n* = 20)
Age (mean ± SD)	40.35 ± 12.29	41.32 ± 10.06	44.37 ± 9.84
Male/female	6/14	8/12	6/12
Symptom score	0	0–2	0–3
Sign score	0	1	2–5
Body mass index (mean ± SD)	21.65 ± 2.85	23.44 ± 3.25	23.83 ± 3.65
Cigarette smoking/alcohol drinking	0	0	0
Number of remaining teeth (mean ± SD)	29.65 ± 2.85	29.05 ± 1.90	29.11 ± 1.70

**TABLE 2:** Demographic and clinical parameters of the tissue samples found no statistically significant differences in age (*P* = 0.090 b) and gender (*P* = 0.800 a) of each group.

Characteristics	Healthy controls (*n* = 8)	Reticular OLP patients (*n* = 12)	Erosive OLP patients (*n* = 12)
Age (mean ± SD)	38.500 ± 13.554	47.429 ± 10.799	36.125 ± 14.126
Male/female	4/4	8/4	5/7
Sites (Buccal mucosa or tongue)	Normal oral mucosa around the tooth extraction wound and sublingual cysts	7/5	8/4

The study inclusion criteria were as follows: (i) 22–62 years of age; (ii) no OLP treatment or prescription drug use at least 2 months before sampling; (iii) no history of antibiotic use at least 1 month before sampling; (iv) no immunomodifier use within 3 months before sampling; and (v) no history of serious systemic disorders.

Individuals were excluded from the study for fulfilling any of the following conditions: (i) other known oral mucosal diseases; (ii) patients with tumor; (iii) lichenoid reactions caused by drugs or amalgam filling; (iv) pregnancy, lactation, and contraceptives; (v) use of any mouthwash within 7 days; (vi) patients diagnosed as periodontitis, presence of visible caries, and removable or fixed dentures.

### Samples Acquisition and Storage

#### Collection of Saliva Samples

Due to the diurnal variation of saliva, saliva samples were collected between 8 a.m. and 12 noon, to minimize the variability in salivary flow and compositions. No consumption of any food or beverage at least 1 h prior to sample collection was necessary before conducting a comprehensive oral examination of all subjects. After gargling, about 2.5 ml of spontaneous, unstimulated whole saliva (UWS) samples was collected through the technique suggested by Navazesh (1993) in a 5-ml sterile DNA-free (RNA-free) conical tube from each subject; coughing was prohibited during the period. All samples were transferred to the laboratory on ice within 3 h, frozen, and stored at −80°C until further processing.

#### Collection of Tissue Samples

The tissue samples of OLP lesions were all from the representative areas of the buccal mucosa or tongue of OLP patients, while the healthy control tissues were obtained from normal oral mucosa around the tooth extraction wound and sublingual cyst. To reduce sample contamination, tissue samples were fixed in 10% neutral-buffered formalin for 24 h within 1 h after surgical resection and paraffin-embedded using standard histology methods. Tissue samples were taken in duplicate; one was cut into 8−μm−thick serial scrolls and placed into a sterile 2-ml centrifuge tube, which was stored separately at −20°C for 16S rDNA sequencing, and the other was taken from six healthy controls, five reticular OLP, and five erosive OLP for FISH and hematoxylin and eosin (H&E) staining.

### DNA Extraction and Polymerase Chain Reaction (PCR) Amplification

Total genomic DNA was extracted from saliva and tissue samples according to the manufacturer’s instructions using the E.Z.N.A.^®^ Soil DNA Kit (Omega Bio-Tek, Norcross, GA, United States). Polymerase chain reaction (PCR) products of all samples were amplified by using the following parameters: denaturation at 95°C for 2 min, followed by 25 cycles of denaturation at 95°C for 30 s, annealing at 55°C for 30 s, elongation at 72°C for 30 s, and incubation at 72°C for 5 min. The V3–V4 region of the bacterial 16S rDNA gene was amplified by PCR using primers 341F: 5′-barcode-CCTAYGGGRBGCASCAG-3′ and 806R: 5′-GGACTACNNGGGTATCTAAT-3′, where the barcode was an eight-base sequence unique to each sample. PCR reactions were performed in triplicate 20-μl mixture containing 4 μl of 5 × FastPfu Buffer, 2 μl of 2.5 mM dNTPs, 0.8 μl of each primer (5 μM), 0.4 μl of FastPfu Polymerase, and 10 ng of template DNA. Amplicons were extracted from 2% agarose gels and purified using the AxyPrep DNA Gel Extraction Kit (Axygen Biosciences, Union City, CA, United States) according to the manufacturer’s instructions and quantified using QuantiFluor^TM^-ST (Promega, United States).

### Library Construction and Sequencing

Purified PCR products were quantified by Qubit^®^3.0 (Life Invitrogen), and every 24 amplicons whose barcodes were different were mixed equally. The pooled DNA product was used to construct the Illumina pair-end library following Illumina’s genomic DNA library preparation procedure. Then, the amplicon library was paired-end sequenced (2 × 300) on an Illumina MiSeq platform (Shanghai BIOZERON Co., Ltd.) according to the standard protocols.

### H&E Staining and FISH on Tissue Sections

Hematoxylin and eosin staining and FISH on tissue sections only included 10 OLP patients and six healthy controls due to insufficient tissue surplus. All formalin-fixed, paraffin-embedded (FFPE) specimens were fixed onto uncoated slides, and the slides were deparaffinized and then hydrated. For each subject, two 4-μm-thick FFPE tissue biopsy sections were prepared, one using standard H&E staining to identify and mark the lesion site of interest, and the other using digoxigenin (DIG)-labeled FISH analysis of 16S rDNA gene universal probe (Lim and Lim, 2017). The specific fluorescence signal of bacterial invasion in the lesion tissue of OLP patients was detected compared with healthy controls. FISH staining was performed as previously described (Moter and Göbel, 2000). Briefly, FFPE tissue sections at 4-μm thickness were fixed on slides, dried in a lab oven with thermostat set to 62°C for 2 h, and then the slices are sequentially placed into xylene I 15 min–xylene II 15 min–anhydrous ethanol I 5 min–anhydrous ethanol II 5 min–85% alcohol 5 min–75% alcohol 5 min; after that, the slides were rinsed with diethyl pyrocarbonate (DEPC) 5 min and immersed in repair solution for 5–10 min. After natural cooling, 20 μg/ml proteinase K was added dropwise for digestion for 1–5 min, and phosphate buffer saline (PBS) was washed for 3 × 5 min after pure water washing. The pre-hybridization solution was added dropwise and incubated at 37°C for 1 h. Discard the pre-hybridization solution, drop the hybridization solution containing the DIG probe, and hybridize it overnight at 37°C in a constant temperature box. Hybridization was performed with unlabeled probes as negative controls. The hybridization solution was washed away, 2 × saline sodium citrate (SSC), 37°C for 10 min, 1 × SSC, 37°C for 2 × 5 min, and 0.5 × SSC for 10 min at room temperature. 4’-6-Diamidino-2-phenylindole (DAPI) counterstain was applied and incubated in the dark for 8 min. After washing, the anti-fluorescence quenched sealer was added dropwise. The nucleus of nucleic acid stain DAPI stained with non-specific staining was blue under the ultraviolet laser, and the representative positive signal carboxy-fluorescein (FAM) (488) was green. The images were captured at a magnification of 200 times and processed with the software Image-pro plus 6.0 (Media Cybernetics, Inc., in Rockville, MD, United States). The positive integrated optical density (IOD) and pixel AREA (AREA) of the epithelial and lamina propria of each image were analyzed, and the average optical density (AOD) of each sample was calculated, AOD = IOD/AREA, the higher AOD, the higher the positive expression level.

### Bioinformatic Analysis

Operational taxonomic units (OTUs) were clustered with 97% similarity cutoff using UPARSE (version 7.1^1^) and chimeric sequences were identified and removed using UCHIME. Afterward, the phylogenetic affiliation of each 16S rDNA gene sequence was blasted against the Silva (SSU132) 16S rDNA database and analyzed by Ribosomal Database Project (RDP) Classifier^2^ using a confidence threshold of 70% (Amato et al., 2013). Finally, an OTU table was obtained and based on the taxonomic information; statistical analysis of the community structure was performed at each classification level.

### Clustering and Statistical Analysis

Usearch (version 10^3^) was the main platform of clustering and statistical analysis. The relative abundance of bacterial taxa in each sample between groups was calculated and analyzed by the Mann–Whitney *U*-test. Mothur v.1.21.1 (Schloss et al., 2009) was conducted to reveal diversity analysis, including the Chao1 index and Shannon index. Beta diversity analysis was performed using UniFrac (Lozupone et al., 2011) to compare the results of principal component analysis (PCA), and the community ecology package, R-forge (Vegan 2.0) was used to generate PCA figures. Linear discriminant analysis effect size (LEfSe) analysis used the Kruskal–Wallis, Wilcoxon rank-sum test, and Spearman’s rank correlation test to examine the changes and dissimilarities among taxa. All statistical analyses were performed using SPSS (version 23.0; SPSS Inc., Chicago, IL, United States). *P* < 0.05 was considered statistically significant.

## Results

The healthy controls (*n* = 4) and the erosive OLP (*n* = 4) from 32 FFPE tissue samples of 16S rDNA gene sequencing did not yield enough DNA to be detected or detectable PCR products after 40 cycles of PCR. These samples were excluded from further statistical analysis, which means that tissue samples, including four healthy controls and 20 OLP patients, were eventually analyzed. In the H&E staining and FISH of tissue samples, 10 OLP patients and six healthy controls were finally selected due to insufficient tissue surplus.

After preprocessing with 60 saliva and 24 tissue samples, 1,923,413 and 969,405 high-quality sequences with an average length of 434.73 and 411.77 bp were obtained, with an average of 32,057 and 40,392 sequences per sample. From these sequences, 22 and 30 known phyla, 111 and 196 known families, as well as 234 and 426 known genera were identified, respectively. A total of 908 and 1299 OTUs were detected, and Good’s estimator of coverage was 99.79 and 99.94%, suggesting that the results of 16S rDNA identified by saliva and tissue libraries in this study could represent the majority of bacterial sequences in the samples.

### Basic Characteristics of Study Subjects

The basic information and characteristics of the study subjects are shown in Tables 1, 2. All samples were tested by Pearson Chi-square test (a) and one-way analysis of variance (b); there was no age or gender bias among each group.

### The Microbiota Profile of Saliva and Tissue Samples

#### Alpha Diversity Analysis (Diversity Within Samples)

**FIGURE 1:**
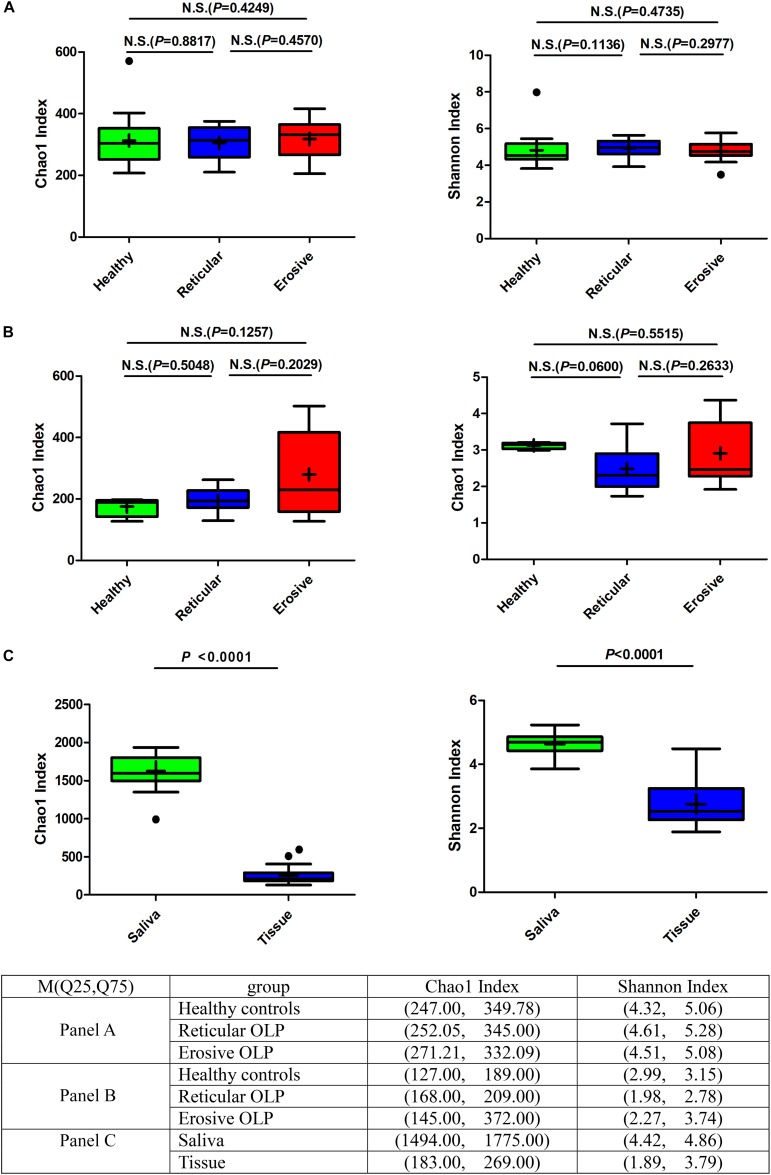
Box plot: Alpha diversity among healthy controls, reticular OLP, and erosive OLP. **(A)** Chao1 index and Shannon index among healthy controls, reticular OLP, and erosive OLP in saliva samples. **(B)** Chao1 index and Shannon index among healthy controls, reticular OLP, and erosive OLP in tissue samples. **(C)** Chao1 index and Shannon index between saliva and tissue samples. Box plots show the top quartile, median, and bottom quartile; “ + ” means the average value and “^∙^” means the outlier. N.S. means no statistical difference. The *P*-value was obtained by the Mann–Whitney *U*-test.

Alpha diversity was used to analyze differences in microbial diversity. The Chao1 index (species richness) and Shannon index (microbial diversity) between the healthy controls, reticular OLP, and erosive OLP in saliva (Figure 1A) and tissue (Figure 1B) samples did not show a significant difference, respectively (*P* > 0.05). However, there were strong variations of the Chao1 index and Shannon index between OLP saliva and tissues (*P* < 0.0001, Figure 1C). The results showed that compared with tissues, the salivary bacterial diversity of OLP patients significantly increased.

#### Principal Component Analysis (PCA) (Diversity Between Samples)

**FIGURE 2:**
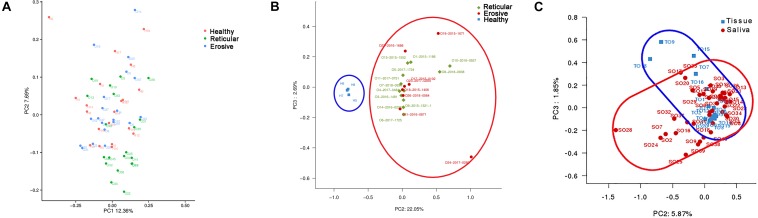
Principal component analysis (PCA) of saliva and tissue microbiota based on the weighted unifrac distance matrix. **(A)** Each point represents reticular OLP (green circle), erosive OLP (blue circle), and healthy controls (red circle) for each saliva sample. PC1 explained 12.36% of the variation, and PC2 explained 7.69% of the variation. **(B)** Each point on behalf reticular OLP (green diamond), erosive OLP (red circle), and healthy controls (blue square) for each tissue sample. PC2 explained 22.05% of the variation, and PC3 explained 2.69% of the variation. **(C)** Each point represents OLP saliva (red circle) and OLP tissue (blue square). PC2 explained 5.87% of the variation, and PC3 explained 1.85% of the variation. Each point represents a sample and is colored by sample type. The more similar samples are, the closer they are in the graph.

The weighted UniFrac phylogenetic distance matrices were used to calculate the beta diversity, indicating the difference in bacterial community structure, which can be seen in Bray–Curtis distance PCA plots (Figure 2). From Figure 2A, the result indicated that there was no obvious separation between healthy controls, reticular OLP and erosive OLP, and there was no statistical difference in the weighted measurement in saliva (*P* = 0.146). However, the OLP group was completely separated from the healthy controls in tissue (Figure 2B), and the weighted measurements of healthy controls and OLP (*P* = 0.004), healthy controls and reticular OLP (*P* = 0.001), and healthy controls and erosive OLP (*P* = 0.002) were also statistically different. Except for reticular OLP and erosive OLP, which has no statistically significant difference in the weighted measurement (*P* = 0.150), this indicates that the overall structure of the bacterial community in the population was significantly different, while the structure of reticular and erosive OLP microbial community was not significantly different. There was a partial overlap between saliva and tissue samples of OLP patients (Figure 2C) and significant difference in the weighted measurement (*P* = 0.001), suggesting that OLP microorganisms between saliva and tissue samples were partially close to each other but still different.

### Changes of Microbiota at the Phylum, Family, and Genus Levels in Saliva and Tissues

**FIGURE 3:**
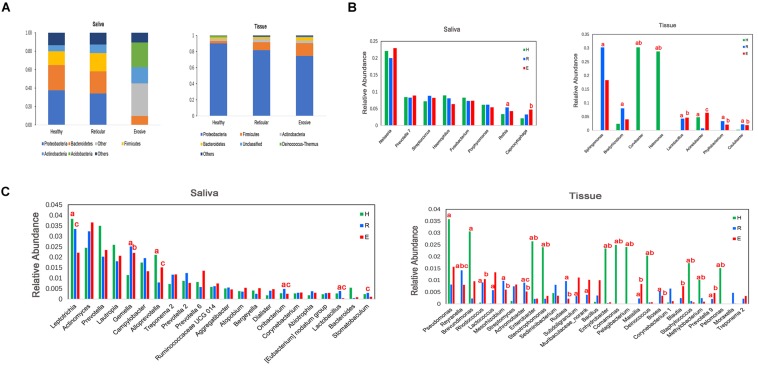
Comparison of average relative abundance of major phyla and genera (>0.2%) in healthy controls (H), reticular OLP (R), and erosive OLP (E) in saliva and tissue samples. **(A)** Comparison of the phylum level among the three groups. **(B)** Comparison among the top eight genera of the three groups. **(C)** Comparison of 9–30 genera of the three groups. a: H vs R; b: H vs E; c: R vs E; red letters indicate *P* < 0.05.

Bacterial communities in OLP and healthy controls were analyzed at different taxonomic levels (Figure 3). Represented phylum in saliva and tissue samples was Proteobacteria. At the family level, the saliva microbiota was dominated by Neisseriaceae, followed by Prevotellaceae, Pasteurellaceae, and so on. Caulobacteraceae was shown to be the major family in tissue samples, followed by Sphingomonadaceae, Burkholderiaceae, and so on. *Neisseria* had the highest average relative abundance at the genus level in saliva samples. Compared with healthy controls, the average relative abundance of 11 genera, such as *Capnocytophaga*, *Gemella*, and *Granulicatella*, was significantly higher in OLP saliva (*P* < 0.05). In the tissue samples, 41 genera such as *Escherichia*–*Shigella*, *Phyllobacterium*, and *Megasphaera* were significantly higher in OLP than those in the healthy controls (*P* < 0.05). In particular, compared with healthy controls, *Phyllobacterium* and *Megasphaera* were not only significantly higher in both reticular and erosive OLP tissues (*P* < 0.05), but also higher in reticular and erosive OLP saliva (*P* > 0.05). In tissue samples, *Escherichia*–*Shigella*, *Bacteroides*, and so on only existed in OLP (*P* < 0.05). At the species level, in the saliva samples, the average relative abundance of *Prevotella saccharolytica JCM 17484*, *Lactobacillus plantarum*, *Lactobacillus* sp. *ARSOA BB*, and *Neorhizobium huautlense* in the OLP was significantly higher than those in the healthy controls (*P* < 0.05). In tissue samples, the average relative abundance of seven known species including *Methylobacterium aquaticum*, *Achromobacter xylosoxidans* subsp. *Xylosoxidans*, *Sphingomonas mali*, *Pseudomonas poae*, *Tsukamurella tyrosinosolvens*, *Rhodopseudomonas pentothenatexigens*, and *Megasphaera elsdenii DSM 20460* was significantly higher in OLP than that in the healthy controls (*P* < 0.05).

### Bacterial Community Structures of Saliva and Tissue Samples

#### Saliva Samples of Healthy Controls and the OLP

**FIGURE 4:**
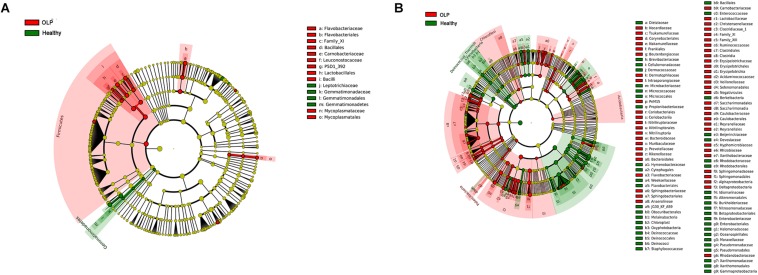
LEfSe analysis cladogram of saliva and tissue samples between the healthy controls and the OLP. **(A)** Saliva samples’ LEfSe analysis cladogram. **(B)** Tissue samples’ LEfSe analysis cladogram. Colored nodes from the inner to the outer rings, respectively, represent the level of phylum (p), class (c), order (o), family (f), and genus (g); only the level of class to family was labeled and annotated on the right side of the cladogram. Differences were indicated by different colors: green for healthy controls, red for OLP, and yellow for non-significant. The diameter of each circle was proportional to the abundance of the taxa.

To further identify the specific bacterial taxa related to OLP, we used the linear discriminant analysis (LEfSe) to compare the microbiota of OLP and healthy controls. Figure 4A shows the greatest difference in taxa between the two groups. Overall, two phyla, two classes, five orders, seven families, and 11 genera were detected to be different from each other. The bacteria that play an important role in OLP with the significant difference in abundance were Firmicutes at the phylum level, Leuconostocaceae, Flavobacteriaceae, Mycoplasmataceae, and so on at family level. At the taxonomic level of the genus, there were *Capnocytophaga*, *Gemella*, *Granulicatella*, and so on.

#### Tissue Samples of Healthy Controls and the OLP

Linear discriminant analysis (LEfSe) was used to determine which taxa could explain the changes observed by UniFrac in the tissue samples (Figure 4B). A total of five phyla, 14 classes, 28 orders, 54 families, and 86 genera were found to be different between the two groups. Acidobacteria, Chloroflexi, and Patescibacteria were significantly enriched in OLP, and at the family level, there were Bacteroidaceae, Flavobacteriaceae, Sphingobacteriaceae, and so on. At the genus level, discriminative genera between health and disease were as follows: in healthy controls, *Streptococcus*, *Micrococcus*, *Sphingobium*, and so on; in OLP, *Escherichia*–*Shigella*, *Megasphaera*, Phyllobacterium, and so on.

### Pearson Correlation Heatmap Analysis of the Bacterial Genera With Average Relative Abundance > 0.2% in Saliva and Tissue Samples Between Healthy Controls and the OLP

**FIGURE 5:**
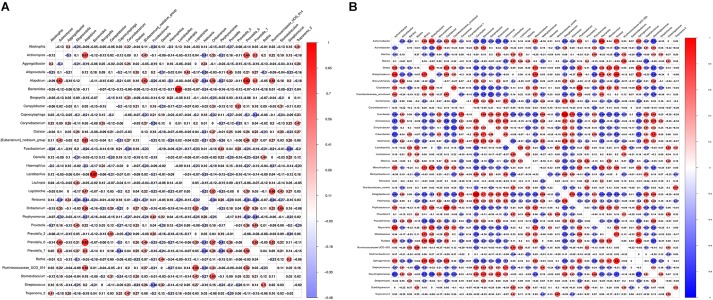
Pearson correlation matrix visualization of saliva and tissue samples between healthy controls and OLP. **(A)** Pearson correlation matrix visualization of saliva healthy controls and OLP with genera average relative abundance > 0.2%. **(B)** Pearson correlation matrix visualization of tissue healthy controls and OLP with genera average relative abundance > 0.2%. In the figure, the larger the circle, the higher the correlation coefficient. The correlation with *P* > 0.01 was left blank; red represented positive correlation and blue represented negative correlation. The correlation value ranged from -1.00 (blue) to 1.00 (red).

From Figure 5A, we found that *Bacteroides* and *Lactobacillus* were the most positively correlated (ρ = 0.87), whereas *Fusobacterium* and *Streptococcus* were the most negatively correlated (ρ = −0.48) in saliva samples between healthy controls and the OLP. However, Pelagibacterium and Curvibacter were the most positively correlated (ρ = 0.99), while Stenotrophomonas and Sphingomoas were the most negatively correlated (ρ = −0.77) in tissue samples between healthy controls and the OLP (Figure 5B).

### FISH of Tissue Samples

**FIGURE 6:**
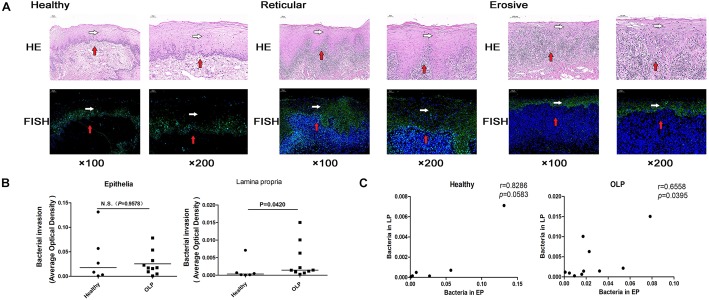
Fluorescence *in situ* hybridization localized bacterial distribution in tissue samples. **(A)** Fluorescence *in situ* hybridization of healthy controls (*n* = 6), reticular OLP (*n* = 5), and erosive OLP (*n* = 5). White arrow represents the epithelia, and red arrow represents the lamina propria. Fluorescent signal FAM (488) was green, and the nucleus was blue. H&E, hematoxylin and eosin staining. FISH, fluorescence *in situ* hybridization. **(B)** Comparison of AOD of bacteria in the epithelial and lamina propria between the healthy controls and the OLP. **(C)** Correlation plots of AOD of bacteria in the epithelial and those in the lamina propria of the healthy controls and the OLP. (*r* and *P* were obtained through Spearman’s rank correlation test). The black bar indicates the median of each group. N.S. means no statistical difference. *P* < 0.05 was obtained by the Mann–Whitney *U*-test.

Fluorescence *in situ* hybridization is an effective approach for locating the spatial distribution of bacteria in tissue samples. In healthy controls, reticular OLP, and erosive OLP, the bacterial signal in the epithelia was stronger than that in the lamina propria. Compared with the healthy controls, the epithelial and lamina propria of reticular and erosive OLP both had strong bacterial signals (Figure 6A). AOD of bacteria in OLP lamina propria was significantly higher than that in healthy controls (*P* = 0.0420, Figure 6B), while no difference was observed in the epithelial (*P* = 0.9578, Figure 6B). There was a significant positive correlation between the AOD within the epithelial and lamina propria of the OLP (*r* = 0.6695, *P* = 0.0342, Figure 6C), but not in the healthy controls (*r* = 0.3214, *P* = 0.4976, Figure 6C).

## Discussion

To date, studies of the OLP addressing microbial communities have been largely focused on OLP patients with mucosal surface swabs (He et al., 2017) and saliva (Wang et al., 2016). Studies on microbial aspects in OLP tissues have only reported *Mycoplasma salivarium* (Mizuki et al., 2017); knowledge about the community structure and composition of bacterial flora in OLP tissues was still lacking. We not only studied the microbial characteristics of the two sample types but also studied the effect of the difference between them on the microbial profiles of the OLP, aiming to determine and characterize the microbiota of OLP saliva and tissues. Moreover, alpha diversity analysis showed that there was no significant difference in microbial richness or diversity in saliva and tissues among healthy controls, reticular OLP, and erosive OLP, respectively, which was in line with the results from Wang et al. (2016) of saliva samples. However, there was a difference in the beta diversity of tissue samples between OLP and healthy controls, representing more inter-group variation.

Our study analyzed bacterial communities in OLP tissues and compared the relationship between the overall oral microenvironment and the microenvironment of the local lesion area. In this paper, we provided evidence that the diversity and composition of microbial communities in OLP saliva and tissue samples were significantly different. Compared with OLP tissues, OLP saliva showed significantly higher alpha diversity. Saliva and tissue samples of OLP patients showed significantly different alpha diversity from each other, suggesting that OLP tissues also contained bacteria from the mucosal surface. We speculated that the bacteria on the mucosal surface of OLP tissues may pass through the damaged epithelium to subepithelial tissue, and over time, these bacteria may develop into a unique bacterial community.

Low tissue microbial diversity may be due to the overgrowth of potential pathogens, which may trigger or exacerbate disease states. Inflammation or immune imbalance caused by OLP may also create an unfavorable growth environment for microorganisms, thus reducing the bacterial diversity of OLP tissues. The microbiota of OLP saliva and tissues was also significantly different. Compared with the healthy controls, *Capnocytophaga*, Gemella, and so on were higher in OLP saliva, while *Escherichia*–*Shigella*, *Megasphaera*, and so on were higher in OLP tissues. This result demonstrated that the differences in the composition of bacterial communities could be attributed to environmental factors and disease susceptibility. Saliva may represent the change of the whole oral microflora, which was susceptible to the influence of oral hygiene and oral diseases. Different microenvironments may be the driving factors for the different bacterial community structures. An alternative explanation for these differences might be homing to saliva samples contains rich planktonic bacteria on the mucosal surface of OLP lesions, while tissue samples contain more intracellular bacteria or biofilm-related bacteria.

Besides, we also found some enrichment of higher abundant taxa in OLP saliva and tissues, including the family Carnobacteriaceae, Flavobacteriaceae, Caulobacteraceae, and Veillonellaceae, as well as the genus *Megasphaera*, *Sphingomonas*, and *Delftia*. Compared with healthy controls, they were significantly higher in OLP tissues, and also higher in OLP saliva, but there was no statistical difference. Yun et al. believed that OLP may be associated with microbial infections (Baek and Choi, 2018), while the high levels of Flavobacteriaceae, Veillonellaceae, *Delftia*, and *Megasphaera* were thought to be associated with inflammation and infectious diseases, such as infective endocarditis (Tomida et al., 2019), bacterial vaginosis (Tabatabaei et al., 2019), pancreatic cancer (Mei et al., 2018), and periodontitis (Kumar et al., 2005).

The pathogenesis of OLP was related to the imbalance of TH1/TH2; Toll-like receptors-2 (TLR2) has been found to promote an inflammatory response by inducing TH1/TH2 imbalance (Wang et al., 2018). Janardhanam et al. (2012) found that TLR2 messenger RNA (mRNA) was significantly lower in OLP epithelial cells and decreased in UWS epithelial cells of OLP patients. In fecal samples from patients with functional gastroenteropathy, Carnobacteriaceae was inversely correlated with the expression of TLR2 (Dong et al., 2017) and correlated with the immune status of leukemia patients (Wang et al., 2014). Besides, Veillonellaceae has been found to be associated with juvenile idiopathic arthritis involving autoimmune diseases, and increased in patients with non-enthesitis-related arthritis (Di Paola et al., 2016) and schistosomiasis (Ajibola et al., 2019) associated with downregulation of TH2 immune response.

This study found that *Megasphaera* was significantly higher in both reticular and erosive OLP tissues than in the healthy controls, and was also higher in reticular and erosive OLP saliva than in the healthy controls. *Megasphaera* may be associated with viral infection (Lu et al., 2017), contributing to host immune defense, such as activating T cell-dependent immune response. Besides that, *Megasphaera*, *Sphingomonas*, and Caulobacteraceae were known to be associated with cancer. For instance, *Sphingomonas* increased in patients with colitis-related cancer (Yu, 2018) and played a dominant role in patients with immunoglobulin A nephropathy (Watanabe et al., 2017), possibly leading to the activation of mucosal immune response due to interaction with potential host genetic factors. *Megasphaera* and Caulobacteraceae could serve as a microbial biomarker for lung cancer (Lee et al., 2016) and breast carcinogenesis (Meng et al., 2018). It is worth noting that the higher levels of the above taxa in the OLP might be thought of as a potential microbial biomarker of the OLP.

Interestingly, when analyzing the tissue samples, we found that *Escherichia*–*Shigella* only existed in the OLP. *Escherichia*–*Shigella* associated with the pro-inflammatory state was increased in ulcerative colitis, and inflammatory state may be related to host immunity–microbial interaction (Xu et al., 2018). The increase of *Escherichia*–*Shigella* in patients with autoimmune disease, hypothyroidism, and Hashimoto’s thyroiditis may be associated with the increase of the serum circulating antibody level (Ishaq et al., 2017). A cross-sectional study by Zucoloto et al. (2019) suggested that the anxiety level was directly related to OLP severity, and Jiang et al. (2018) reported that excessive growth of *Escherichia*–*Shigella* in patients with a generalized anxiety disorder may exacerbate anxiety, and the resulting exotoxins could contribute to inflammatory conditions (Maes et al., 2007).

There was no statistical difference in saliva for health-related *Streptococcus*, while *Streptococcus* was significantly higher in tissue samples from healthy controls. He et al. (2017); Wang et al. (2016), and Yun et al. (Choi et al., 2016) have also reported the same findings in saliva and the mucosal surface. *Streptococcus salivarius*, as an ideal candidate for oral probiotics (Wescombe et al., 2009), did not initiate infection in healthy individuals and may help to establish immune homeostasis and regulate host inflammatory response. In saliva, we found that *Fusobacterium* and *Streptococcus* were the most negatively correlated in the Pearson correlation matrix. This result was consistent with He et al. (2017), who found a significant increase of *Fusobacterium* and a decrease of *Streptococcus* on the buccal mucous surface of the OLP.

By FISH, we found that the AOD of bacteria in the lamina propria of OLP tissues was significantly higher than that of the healthy controls, and the AOD of bacteria in the epithelial and lamina propria of OLP was significantly positively correlated. It suggested that the epithelial barrier function was impaired in local lesions of OLP, and bacteria could invade into the lamina propria through the epithelia of OLP tissues. Notably, this finding was consistent with the results of the *in situ* hybridization of Yun et al. (Choi et al., 2016) using a universal probe targeting bacterial 16S rRNA labeled with DIG. However, 16S rDNA sequencing did not amplify bacterial rDNA in some tissue samples, which may be related to the low bacterial biomass in some tissue samples. FISH had higher accuracy than 16S rDNA sequencing and was not easily affected by pollutants. Moreover, FISH was not only simple, rapid, and inexpensive, but also able to directly locate and identify biofilm in tissues, with higher reliability (Frickmann et al., 2017).

Our current study still has several limitations. First, a small sample size we studied may be insufficient to detect slight differences between groups, so further in-depth studies should be confirmed on a larger cohort to obtain more accurate and comprehensive findings. Second, due to ethical limitations, we could not sample the buccal mucosa or tongue of the “healthy” non-disease control population, so we had to collect the normal oral mucosa tissues around the tooth extraction wound and sublingual cyst of healthy controls. In fact, the PCR amplification of the 16S gene especially in the case of some tissue samples proved relatively difficult; Joss et al. (2015) failed to generate sufficient 16S amplicon from three of 22 subjects by improving PCR amplicon yield and the overall yield of 16S amplicon. Liu et al. (2018) found that 14 of the 16 negative controls failed amplification and bacterial DNA could not be detected. Abreu et al. (2012) also encountered a similar problem. However, PCR amplification of the 16S gene in the FFPE tissue samples was more difficult. Joss et al. (2015) also suggested that the failure to detect 16S amplicon of bacteria could not exclude other biological processes such as viruses and archaea that may be involved in the occurrence and development of diseases. Finally, the sensitivity of FISH was lower than PCR, and the presence of autofluorescent particles in certain tissues may produce false-positive results (Prudent and Raoult, 2019). Therefore, it is necessary to further study the species and gene expression differences of the microbiome through macro transcriptome and other research methods.

## Conclusion

In conclusion, our results suggest that the diversity and composition of microbial communities in tissues and saliva samples from the OLP are different, and the presence of the above unusual taxa is mostly related to immune status and inflammation that may worsen the local microenvironment of the OLP. On the other hand, the role of microorganisms in the occurrence and development of diseases lies in the imbalance in the microbial community composition, rather than their existence. Study the specific bacteria in the diseased tissues of OLP patients and how they play an important role in the pathogenesis and development of the disease, which may be a biological approach to prevent OLP.

## Data Availability Statement

The datasets used and/or analyzed during the current study are available from the corresponding author on reasonable request. The raw reads for all samples used in this study have been deposited into the NCBI Sequence Read Archive (SRA) database (accession numbers: SRP216171 and SRP215349).

## Ethics Statement

The studies involving human participants were reviewed and approved by the Ethical Committee of Affiliated Hospital of Stomatology, Nanjing Medical University (permission number PJ2016-034-001) and the Institutional Review Board of Nanjing Medical University (permission number 2014-132). The patients/participants provided their written informed consent to participate in this study.

## Author Contributions

YF conceived and designed the research. XW, ZZ, and NT performed the experiments. XW, NT, and ZZ processed and analyzed the data with help from JZ. YZ, JX, LQ, and LL recruited volunteers and collected samples. XW drafted the manuscript with inputs from all authors.

## Conflict of Interest

The authors declare that the research was conducted in the absence of any commercial or financial relationships that could be construed as a potential conflict of interest.

## Footnotes

1 http://drive5.com/uparse/

2 http://rdp.cme.msu.edu/

3 http://drive5.com/uparse/

## References

[B1] Abreu N. A. Nagalingam N. A. Song Y. Roediger F. C. Pletcher S. D. Goldberg A. N. (2012). Sinus microbiome diversity depletion and *Corynebacterium tuberculostearicum* enrichment mediates rhinosinusitis. *Sci. Transl. Med.* 4:151ra24. 10.1126/scitranslmed.3003783 22972842 PMC4786373

[B2] Ajibola O. Rowan A. D. Ogedengbe C. O. Mshelia M. B. Cabral D. J. Eze A. A. (2019). Urogenital schistosomiasis is associated with signatures of microbiome dysbiosis in Nigerian adolescents. *Sci. Rep.* 9:829. 10.1038/s41598-018-36709-1 30696838 PMC6351658

[B3] Alexander K. L. Targan S. R. Elson C. O. (2014). Microbiota activation and regulation of innate and adaptive immunity. *Immunol. Rev.* 260 206–220. 10.1111/imr.12180 24942691 PMC4080089

[B4] Alrashdan M. S. Cirillo N. McCullough M. (2016). Oral lichen planus: a literature review and update. *Arch. Dermatol. Res.* 308 539–551. 10.1007/s00403-016-1667-2 27349424

[B5] Amato K. R. Yeoman C. J. Kent A. Righini N. Carbonero F. Estrada A. (2013). Habitat degradation impacts black howler monkey (*Alouatta pigra*) gastrointestinal microbiomes. *ISME J.* 7 1344–1353. 10.1038/ismej.2013.16 23486247 PMC3695285

[B6] Baek K. Choi Y. (2018). The microbiology of oral lichen planus: is microbial infection the cause of oral lichen planus? *Mol. Oral Microbiol.* 33 22–28. 10.1111/omi.12197 28869787

[B7] Choi Y. S. Kim Y. Yoon H. J. Baek K. J. Alam J. Park H. K. (2016). The presence of bacteria within tissue provides insights into the pathogenesis of oral lichen planus. *Sci. Rep.* 6:29186. 10.1038/srep29186 27383402 PMC4935860

[B8] Crincoli V. Di Bisceglie M. B. Scivetti M. Lucchese A. Tecco S. Festa F. (2011). Oral lichen planus: update on etiopathogenesis, diagnosis and treatment. *Immunopharmacol. Immunotoxicol.* 33 11–20. 10.3109/08923973.2010.498014 20604639

[B9] Di Paola M. Cavalieri D. Albanese D. Sordo M. Pindo M. Donati C. (2016). Alteration of fecal microbiota profiles in juvenile idiopathic arthritis. associations with HLA-B27 allele and disease status. *Front. Microbiol.* 7:1703. 10.3389/fmicb.2016.01703 27833598 PMC5080347

[B10] Dong L. N. Wang J. P. Liu P. Yang Y. F. Feng J. Han Y. (2017). Faecal and mucosal microbiota in patients with functional gastrointestinal disorders: correlation with toll-like receptor 2/toll-like receptor 4 expression. *World J. Gastroenterol.* 23 6665–6673. 10.3748/wjg.v23.i36.6665 29085211 PMC5643287

[B11] Farhi D. Dupin N. (2010). Pathophysiology, etiologic factors, and clinical management of oral lichen planus, part I: facts and controversies. *Clin. Dermatol.* 28 100–108. 10.1016/j.clindermatol.2009.03.004 20082959

[B12] Frickmann H. Zautner A. E. Moter A. Kikhney J. Hagen R. M. Stender H. (2017). Fluorescence in situ hybridization (FISH) in the microbiological diagnostic routine laboratory: a review. *Crit. Rev. Microbiol.* 43 263–293. 10.3109/1040841x.2016.1169990 28129707

[B13] Gao L. Xu T. Huang G. Jiang S. Gu Y. Chen F. (2018). Oral microbiomes: more and more importance in oral cavity and whole body. *Protein Cell* 9 488–500. 10.1007/s13238-018-0548-1 29736705 PMC5960472

[B14] He Y. Gong D. Shi C. Shao F. Shi J. Fei J. (2017). Dysbiosis of oral buccal mucosa microbiota in patients with oral lichen planus. *Oral Dis.* 23 674–682. 10.1111/odi.12657 28199766

[B15] Ishaq H. M. Mohammad I. S. Guo H. Shahzad M. Hou Y. J. Ma C. (2017). Molecular estimation of alteration in intestinal microbial composition in Hashimoto’s thyroiditis patients. *Biomed. Pharmacother.* 95 865–874. 10.1016/j.biopha.2017.08.101 28903182

[B16] Janardhanam S. B. Prakasam S. Swaminathan V. T. Kodumudi K. N. Zunt S. L. Srinivasan M. (2012). Differential expression of TLR-2 and TLR-4 in the epithelial cells in oral lichen planus. *Arch. Oral Biol.* 57 495–502. 10.1016/j.archoralbio.2011.10.013 22119043

[B17] Jiang H. Y. Zhang X. Yu Z. H. Zhang Z. Deng M. Zhao J. H. (2018). Altered gut microbiota profile in patients with generalized anxiety disorder. *J. Psychiatr. Res.* 104 130–136. 10.1016/j.jpsychires.2018.07.007 30029052

[B18] Joss T. V. Burke C. M. Hudson B. J. Darling A. E. Forer M. Alber D. G. (2015). Bacterial communities vary between sinuses in chronic rhinosinusitis patients. *Front. Microbiol.* 6:1532. 10.3389/fmicb.2015.01532 26834708 PMC4722142

[B19] Kramer I. R. Lucas R. B. Pindborg J. J. Sobin L. H. (1978). Definition of leukoplakia and related lesions: an aid to studies on oral precancer. *Oral Surg. Oral Med. Oral Pathol.* 46 518–539. 280847

[B20] Kumar P. S. Griffen A. L. Moeschberger M. L. Leys E. J. (2005). Identification of candidate periodontal pathogens and beneficial species by quantitative 16S clonal analysis. *J. Clin. Microbiol.* 43 3944–3955. 16081935 10.1128/JCM.43.8.3944-3955.2005 PMC1233920

[B21] Lee S. H. Sung J. Y. Yong D. Chun J. Kim S. Y. Song J. H. (2016). Characterization of microbiome in bronchoalveolar lavage fluid of patients with lung cancer comparing with benign mass like lesions. *Lung Cancer* 102 89–95. 10.1016/j.lungcan.2016.10.016 27987594

[B22] Lim A. S. Lim T. H. (2017). Fluorescence in situ hybridization on tissue sections. *Methods Mol. Biol.* 1541 119–125. 27910019 10.1007/978-1-4939-6703-2_11

[B23] Liu Y. Wong K. K. Ko E. Y. Chen X. Huang J. Tsui S. K. (2018). Systematic comparison of bacterial colonization of endometrial tissue and fluid samples in recurrent miscarriage patients: implications for future endometrial microbiome studies. *Clin. Chem.* 64 1743–1752. 10.1373/clinchem.2018.289306 30237148

[B24] Lozupone C. Lladser M. E. Knights D. Stombaugh J. Knight R. (2011). UniFrac: an effective distance metric for microbial community comparison. *ISME J.* 5 169–172. 10.1038/ismej.2010.133 20827291 PMC3105689

[B25] Lu H. F. Li A. Zhang T. Ren Z. G. He K. X. Zhang H. (2017). Disordered oropharyngeal microbial communities in H7N9 patients with or without secondary bacterial lung infection. *Emerg. Microb. Infect.* 6:e112. 10.1038/emi.2017.101 29259328 PMC5750457

[B26] Maes M. Mihaylova I. Leunis J. C. (2007). Increased serum IgA and IgM against LPS of enterobacteria in chronic fatigue syndrome (CFS): indication for the involvement of gram-negative enterobacteria in the etiology of CFS and for the presence of an increased gut-intestinal permeability. *J. Affect. Disord.* 99 237–240. 17007934 10.1016/j.jad.2006.08.021

[B27] Masaki M. Sato T. Sugawara Y. Sasano T. Takahashi N. (2011). Detection and identification of non-Candida albicans species in human oral lichen planus. *Microbiol. Immunol.* 55 66–70. 10.1111/j.1348-0421.2010.00285.x 21175776

[B28] Mei Q. X. Huang C. L. Luo S. Z. Zhang X. M. Zeng Y. Lu Y. Y. (2018). Characterization of the duodenal bacterial microbiota in patients with pancreatic head cancer vs. healthy controls. *Pancreatology* 18 438–445. 10.1016/j.pan.2018.03.005 29653723

[B29] Meng S. Chen B. Yang J. Wang J. Zhu D. Meng Q. (2018). Study of microbiomes in aseptically collected samples of human breast tissue using needle biopsy and the potential role of tissue microbiomes for promoting malignancy. *Front. Oncol.* 8:318. 10.3389/fonc.2018.00318 30175072 PMC6107834

[B30] Mizuki H. Abe R. Kogi S. Mikami T. (2017). Immunohistochemical detection of Mycoplasma salivarium in oral lichen planus tissue. *J. Oral Pathol. Med.* 46 649–656. 10.1111/jop.12568 28295632 PMC5600092

[B31] Mostafa D. Tarakji B. (2015). Photodynamic therapy in treatment of oral lichen planus. *J. Clin. Med. Res.* 7 393–399. 10.14740/jocmr2147w 25883701 PMC4394911

[B32] Moter A. Göbel U. B. (2000). Fluorescence in situ hybridization (FISH) for direct visualization of microorganisms. *J. Microbiol. Methods* 41 85–112. 10991623 10.1016/s0167-7012(00)00152-4

[B33] Navazesh M. (1993). Methods for collecting saliva. *Ann. N. Y. Acad. Sci.* 694 72–77. 8215087 10.1111/j.1749-6632.1993.tb18343.x

[B34] Olson M. A. Rogers R. S. Bruce A. J. (2016). Oral lichen planus. *Clin. Dermatol.* 34 495–504. 10.1016/j.clindermatol.2016.02.023 27343965

[B35] Prudent E. Raoult D. (2019). Fluorescence in situ hybridization, a complementary molecular tool for the clinical diagnosis of infectious diseases by intracellular and fastidious bacteria. *FEMS Microbiol. Rev.* 43 88–107. 10.1093/femsre/fuy040 30418568

[B36] Schloss P. D. Westcott S. L. Ryabin T. Hall J. R. Hartmann M. Hollister E. B. (2009). Introducing mothur: open-source, platform-independent, community-supported software for describing and comparing microbial communities. *Appl. Environ. Microbiol.* 75 7537–7541. 10.1128/aem.01541-09 19801464 PMC2786419

[B37] Scully C. Carrozzo M. (2008). Oral mucosal disease: lichen planus. *Br. J. Oral Maxillof. Surg.* 46 15–21. 10.1016/j.bjoms.2007.07.199 17822813

[B38] Tabatabaei N. Eren A. M. Barreiro L. B. Yotova V. Dumaine A. Allard C. (2019). Vaginal microbiome in early pregnancy and subsequent risk of spontaneous preterm birth: a case-control study. *BJOG* 126 349–358. 10.1111/1471-0528.15299 29791775

[B39] Tampa M. Caruntu C. Mitran M. Mitran C. Sarbu I. Rusu L. C. (2018). Markers of oral lichen planus malignant transformation. *Dis. Markers* 2018:1959506. 10.1155/2018/1959506 29682099 PMC5846459

[B40] Tomida J. Fujiwara N. Naka T. Morita Y. Sawabe E. Tojo N. (2019). *Spodiobacter cordis* gen. nov. sp. nov., a member of the family Flavobacteriaceae isolated from patients with infective endocarditis. *Microbiol. Immunol.* 63 111–118. 10.1111/1348-0421.12673 30817020

[B41] van der Meij E. H. van der Waal I. (2003). Lack of clinicopathologic correlation in the diagnosis of oral lichen planus based on the presently available diagnostic criteria and suggestions for modifications. *J. Oral Pathol. Med.* 32 507–512. 12969224 10.1034/j.1600-0714.2003.00125.x

[B42] Wang K. Lu W. Tu Q. Ge Y. He J. Zhou Y. (2016). Preliminary analysis of salivary microbiome and their potential roles in oral lichen planus. *Sci. Rep.* 6:22943. 10.1038/srep22943 26961389 PMC4785528

[B43] Wang L. Wu W. Chen J. Li Y. Xu M. Cai Y. (2018). MicroRNA microarray-based identification of involvement of miR-155 and miR-19a in development of oral lichen planus (OLP) by modulating Th1/Th2 balance via targeting eNOS and toll-like receptor 2 (TLR2). *Med. Sci. Monit.* 24 3591–3603. 10.12659/msm.907497 29813046 PMC6003260

[B44] Wang Y. Xue J. Zhou X. You M. Du Q. Yang X. (2014). Oral microbiota distinguishes acute lymphoblastic leukemia pediatric hosts from healthy populations. *PLoS One* 9:e102116. 10.1371/journal.pone.0102116 25025462 PMC4099009

[B45] Watanabe H. Goto S. Mori H. Higashi K. Hosomichi K. Aizawa N. (2017). Comprehensive microbiome analysis of tonsillar crypts in IgA nephropathy. *Nephrol. Dial. Transpl.* 32 2072–2079. 10.1093/ndt/gfw343 27683270

[B46] Wescombe P. A. Heng N. C. Burton J. P. Chilcott C. N. Tagg J. R. (2009). Streptococcal bacteriocins and the case for Streptococcus salivarius as model oral probiotics. *Future Microbiol.* 4 819–835. 10.2217/fmb.09.61 19722837

[B47] Xu J. Chen N. Wu Z. Song Y. Zhang Y. Wu N. (2018). 5-aminosalicylic acid alters the gut bacterial microbiota in patients with ulcerative colitis. *Front. Microbiol.* 9:1274 10.3389/fmicb.2018.01274 PMC6008376 29951050

[B48] Yu L. C. (2018). Microbiota dysbiosis and barrier dysfunction in inflammatory bowel disease and colorectal cancers: exploring a common ground hypothesis. *J. Biomed. Sci.* 25:79. 10.1186/s12929-018-0483-8 30413188 PMC6234774

[B49] Zucoloto M. L. Shibakura M. E. W. Pavanin J. V. Garcia F. T. da Silva Santos P. S. Maciel A. P. (2019). Severity of oral lichen planus and oral lichenoid lesions is associated with anxiety. *Clin. Oral Investig.* 23 4441–4448. 10.1007/s00784-019-02892-2 30989337

