# Corrigendum: Microbial Community Analysis of Saliva and Biopsies in Patients With Oral Lichen Planus

**DOI:** 10.3389/fmicb.2020.01282

**Published:** 2020-06-26

**Authors:** Xuewei Wang, Zhibai Zhao, Nan Tang, Yuping Zhao, Juanyong Xu, Liuyang Li, Ling Qian, Junfeng Zhang, Yuan Fan

**Affiliations:** ^1^Jiangsu Key Laboratory of Oral Diseases, Nanjing Medical University, Nanjing, China; ^2^Department of Oral Medicine, Affiliated Hospital of Stomatology, Nanjing Medical University, Nanjing, China; ^3^Medical School, Nanjing University of Chinese Medicine, Nanjing, China

**Keywords:** OLP, 16S rDNA gene, saliva, tissue, microbiome, fluorescence *in situ* hybridization

In the original article, there was a mistake in the legend for **Figure 1** as published. The statement “Scatter Diagram and Box Plot expressed the same meaning” was incorrect as there was no Scatter Diagram in the article. The correct legend appears below.

**Figure 1**. Box plot: Alpha diversity among healthy controls, reticular OLP, and erosive OLP. **(A)** Chao1 index and Shannon index among healthy controls, reticular OLP, and erosive OLP in saliva samples. **(B)** Chao1 index and Shannon index among healthy controls, reticular OLP, and erosive OLP in tissue samples. **(C)** Chao1 index and Shannon index between saliva and tissue samples. Box plots show the top quartile, median, and bottom quartile; “+” means the average value and “∙” means the outlier. N.S. means no statistical difference. The *P*-value was obtained by the Mann–Whitney *U*-test.

The authors apologize for this error and state that this does not change the scientific conclusions of the article in any way. The original article has been updated.

